# Association between papillary thyroid carcinoma and cervical lymph node metastasis based on ultrasonic radio frequency signals

**DOI:** 10.1002/cam4.6107

**Published:** 2023-05-18

**Authors:** Liuhua Zhou, Yi Zheng, Jincao Yao, Liyu Chen, Dong Xu

**Affiliations:** ^1^ Zhejiang Chinese Medical University Hangzhou China; ^2^ Department of Diagnostic Ultrasound Imaging & Interventional Therapy, Zhejiang Cancer Hospital, Hangzhou Institute of Medicine (HIM), Chinese Academy of Sciences Hangzhou China; ^3^ Key Laboratory of Head & Neck Cancer Translational Research of Zhejiang Province Hangzhou China; ^4^ Taizhou Key Laboratory of Minimally Invasive Interventional Therapy & Artificial Intelligence, Taizhou Branch of Zhejiang Cancer Hospital (Taizhou Cancer Hospital) Taizhou China

**Keywords:** cervical lymph node metastasis, papillary thyroid carcinoma, TI‐RADS, ultrasonic radio‐frequency

## Abstract

**Objective:**

Papillary thyroid carcinoma (PTC) has a high propensity for cervical lymph node metastasis (CLNM). We evaluated the association between PTC radio frequency (RF) signals and CLNM.

**Methods:**

Patients with PTC (*n* = 170) confirmed by pathology after thyroidectomy between July 2019 and May 2022 were enrolled in this retrospective cohort study. Patients were divided into positive and negative groups according to CLNM. Univariate analysis was performed to predict CLNM and a receiver operating characteristic (ROC) curve was generated to evaluate the diagnostic performance of RF signals and the Thyroid imaging Reporting and Data System.

**Results:**

Of 170 patients with 182 nodules included in the study, 11 had multiple nodules. Univariate analysis showed that age, maximum tumor diameter, cross‐sectional and longitudinal aspect ratio, RF quantitative parameters (cross‐sectional intercept, mid‐band, S1, and S4, and longitudinal Higuchi, slope, intercept, mid‐band, S1), and echogenic foci were independently associated with CLNM (*p* < 0.05). The area under the curve (AUC) values of the maximum tumor diameter, longitudinal slope, and echogenic foci were 0.68, 0.61, and 0.62, respectively. Linear regression analysis of maximum tumor diameter, longitudinal slope, and echogenic foci showed that the correlations between longitudinal slope and CLNM were greater than that of echogenic foci (*ß* = 0.203 vs. *ß* = 0.154).

**Conclusion:**

Longitudinal slope and echogenic foci have similar diagnostic efficacy for predicting the risk of CLNM in PTC, although longitudinal slope has a greater correlation with CLNM.

## 1 BACKGROUND

Papillary thyroid carcinoma (PTC) is the most common pathological type of thyroid carcinoma, accounting for approximately 80% of thyroid carcinomas.^1^, ^2^ Although early and timely surgery leads to a better prognosis, PTC is prone to cervical lymph node metastasis (CLNM) and has a high risk of postoperative recurrence and distant metastasis.^3^ Ultrasonography is the primary method for the examination of the thyroid gland; however, the rate of detection of cervical lymph nodes, especially in the central region, is only 18.8%–31.0% because of interference from tracheal, esophageal, and thyroid diseases.^4^, ^5^ Establishing correlations between the ultrasonographic features of PTC and CLNM can help predict the risk of lymph node metastasis preoperatively, thereby providing a basis for developing treatment plans and assessing disease prognosis.^6^, ^7^, ^8^

In the past, assessment of the ultrasonographic features of PTC mainly relied on gray‐scale images. The Thyroid imaging Reporting and Data System (TI‐RADS) standardizes the classification and diagnostic criteria of thyroid nodules and is an effective guide for determining nodule properties. TI‐RADS not only standardizes the differentiation of benign and malignant thyroid nodules, but also has clinical value for the prediction of CLNM in malignant nodules.^9^, ^10^ However, its diagnostic accuracy is related to the experience of the operators and the resolution of the instruments,^11^ which limits its clinical applications.

Ultrasonic radio‐frequency (RF) signals are echo signals generated by the interaction between ultrasound and human tissues; without image post‐processing, such as filtering and smoothing, they can reflect the propagation of ultrasound pulse more realistically.^12^, ^13^ RF has applications in fatty liver disease grading^14^ as well as in the assessment of benign and malignant breast and thyroid nodules.^15^, ^16^ RF signals provide more information than conventional ultrasound images and are more likely to be associated with artificial intelligence (AI). The information from RF signals can be used for machine learning.^17^ Therefore, the combination of RF and AI may be useful for establishing an accurate and efficient diagnosis system for thyroid nodules as well as for thyroid ultrasound screening. For the vast population of thyroid diseases, it could be the first defense to predict the risk of lymph node metastasis before surgery. Through the summary and use of ultrasound examination methods, we can better identify high risk populations, and then determine surgical methods by further radiological examinations.

To the best of our knowledge, this is the first study assessing the correlation between RF signals and CLNM in PTC. The objective of this study was to evaluate the diagnostic efficacy of quantitative RF parameters to lay the foundation for subsequent research on the combination of RF signals and AI.

## 2 MATERIALS AND METHODS

### 2.1 Study subjects

Patients who underwent thyroidectomy in Zhejiang Cancer Hospital between July 2019 and May 2022 were enrolled. Inclusion criteria were as follows: ① first thyroid surgery; ② preoperative papillary thyroid cancer confirmed by puncture; ③ preoperative enhanced CT assessment of neck lymph nodes and distant metastases, and ultrasound examination of the thyroid and neck; and ④ complete ultrasound data available (ultrasound images and videos of RF signals). Exclusion criteria were as follows: ① pulmonary or other distant metastases; ② incomplete postoperative pathological data; ③ unclear and incomplete imaging data. All patients underwent unilateral thyroidectomy or total thyroidectomy. Lateral cervical lymph node dissection was performed in patients with suspected lateral cervical lymph node metastases on comprehensive preoperative assessment and confirmed by puncture^18^, ^19^; all patients underwent prophylactic central lymph node dissection,^20^ and lymph node metastasis was confirmed by postoperative pathology. The study was approved by the Ethics Committee of Zhejiang Cancer Hospital, and all patients included in this study provided their written informed consent.

**FIGURE 1:**
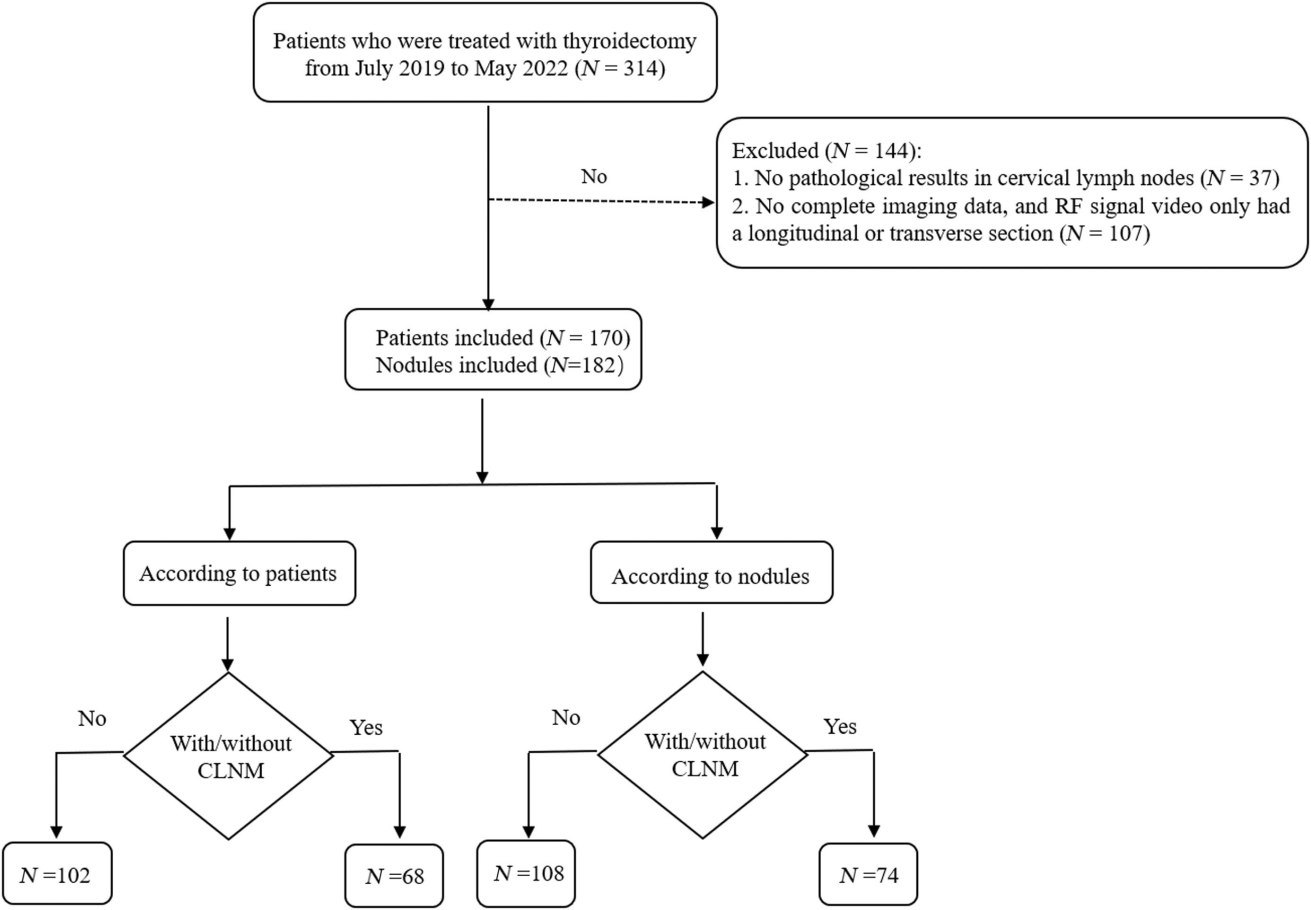
Flow diagram of the study selection procedure.

Of 314 patients who underwent thyroidectomy and were initially included in the study, 144 were excluded because of the following reasons: 37 cases lacked information on CLNM in the postoperative pathological findings, and 107 cases had incomplete imaging data (videos of RF signals containing only a longitudinal or transverse section). Finally, 170 cases with 182 nodes were included in the study, as shown in Figure 1.

### 2.2 Instrument

The GE Logiq E9 (General Electric Healthcare) with a high frequency line array probe (ML6‐15) was used.

### 2.3 The principle of ultrasonic RF signals

**FIGURE 2:**
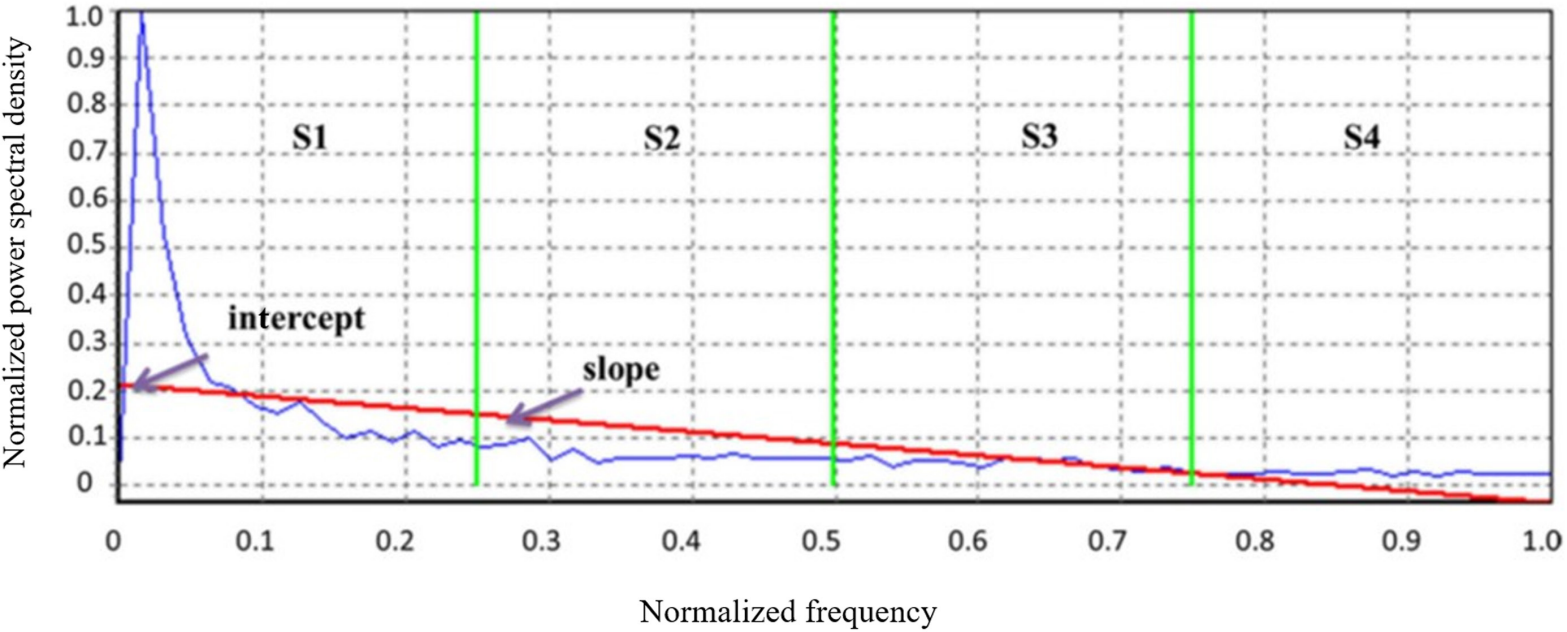
Linear regression fitting for all RF signals within the ROI.

The analysis of RF signals was performed using GE (General Electric Healthcare) software. A region of interest (ROI) was defined, and Fourier transform on each RF signal was executed and normalized; eight spectral feature parameters were generated using the Higuchi fractal dimension based on the Higuchi algorithm. A linear regression fit (red line in Figure 2) was performed for the normalized power spectrum of the RF signal within the ROI (blue curve in Figure 2) to obtain three characteristics: slope, intercept, and mid‐band. Intercept was the intercept of the fitted regression line (i.e., the value of the vertical axis at the intersection of the red line and the vertical axis in Figure 2), and slope was the slope of the regression line.

S1, S2, S3, and S4 are the mean integrals of the four equal parts of the normalized spectra: *k* = 1 ~ *N*/8, *k* = (1 + *N*/8) ~ *N*/4, *k* = (1 + *N*/4) ~ 3 *N*/8, and *k* = (1 + 3 *N*/8) ~ *N*/2, respectively, and the mean values of the power within each band were calculated as follows (S1, S2, S3, and S4):

$$S1=\sum_{k=1}^{N/8}\left|{\overset{⌢}{X}}_{\mathrm{ROI}}\left[k\right]\right|$$

$$S2=\sum_{k=N/8+1}^{N/4}\left|{\overset{⌢}{X}}_{\mathrm{ROI}}\left[k\right]\right|$$

$$S3=\sum_{k=N/4+1}^{3N/8}\left|{\overset{⌢}{X}}_{\mathrm{ROI}}\left[k\right]\right|$$

$$S4=\sum_{k=3N/8+1}^{N/2}\left|{\overset{⌢}{X}}_{\mathrm{ROI}}\left[k\right]\right|$$

Equation: $\left|{\overset{⌢}{X}}_{\mathrm{ROI}}\left[k\right]\right|=\left|{\bar{X}}_{\mathrm{ROI}}\left[k\right]\right|/\max\left(\left|{\bar{X}}_{\mathrm{ROI}}\right|\right)$, the average power spectrum ${\left|\bar{X}\right|}_{\mathrm{ROI}}$ of |*X*[*k*]| (*k* = 1, …, *N*/2) was calculated for all pixel points within the ROI, and the maximum power in the average power spectrum was set to 1; the other components varied equally, and the average power spectrum was normalized.

### 2.4 Acquisition and analysis of ultrasound data

1. Acquisition of ultrasound data

The patients were placed in a supine position with the head tilted back to expose the neck, and multi‐sectional examination of the thyroid gland and bilateral neck was performed. Two sonographers (with more than 10 years of thyroid ultrasound experience) were responsible for the acquisition of ultrasound data. All patients were examined by gray scale, color Doppler, and elastography, and the images were recorded at the workstation. The imaging parameters were adjusted according to the instrument and patient to achieve optimal imaging of the thyroid.

The acquisition parameters of RF signals were set to second harmonic mode, transmit frequency 5 MHz, and receive frequency 10 MHz. Other parameters (mechanical index, imaging depth, gain, etc.) were set according to the diagnostic requirements. The sampling rate was 50 MHz, 312 lines per frame, and 20 frames were acquired for each case. To ensure a consistent sampling data format, thyroid nodules were placed in the middle area of the image to complete the acquisition as much as possible.

2. Analysis of ultrasound data

Ultrasound images were analyzed by two other sonographers (with more than 10 years of ultrasound diagnostic experience) under double‐blind conditions. For the measured values, if the error between the two doctors was within 2 mm, the average value was recorded; if the error exceeded 2 mm or there were other inconsistencies, the senior doctor (with more than 20 years of ultrasound diagnostic experience) reviewed the final decision. The three diameters (length, height, and width), location, composition, echogenicity, aspect ratio, borders, echogenic foci, and other ultrasonographic features of the nodules were recorded. All patients were followed‐up until they underwent thyroidectomy and postoperative pathology results were obtained, with a follow‐up period of 1 week–2 months.

Clinical information included gender and age, and 55 years was selected as the cut‐off age for the TNM staging system for thyroid cancer according to the American Joint Committee on Cancer, 8th edition.^21^ Ultrasound measurements included tumor maximum diameter, tumor volume, cross‐sectional aspect ratio, and longitudinal aspect ratio.

The three diameters of the nodule were defined as follows: (1) a longitudinal scan of the thyroid was completed, the largest section of the nodule was selected, and the longest diameter was defined as the length; (2) the vertical diameter of the longest diameter was defined as the height; and (3) a transverse scan of the thyroid was completed, the largest section of the nodule was selected, and the largest diameter from left to right was defined as the width. Volume of the nodule = 0.523 × length × height × width. Cross‐sectional aspect ratio = height/width and longitudinal aspect ratio = height/length.

Ultrasonographic features were based on TI‐RADS proposed by the American College of Radiology in 2017. Color Doppler visualization of blood flow in thyroid nodules was graded according to Adler's semi‐quantitative criteria.^22^ Elastography was used to measure the stiffness of thyroid nodules, which was graded according to the modified 5‐point scale proposed by Bao‐Ming Luo et al.^23^ as follows: 1: the whole or most of the lesion shows as green; 2: the center of the lesion is blue and the periphery is green; 3: the ratio of green to blue within the lesion is small; 4: the whole lesion is blue; and 5: the area within and around the lesion is blue.

A typical frame of the thyroid nodule and one frame before and after were selected from RF grayscale images, and three grayscale frames were used to analyze RF quantitative parameters. Two sonographers manually outlined the ROI on each frame and selected a rectangular area tangential to the thyroid nodule that contained the nodule and as little surrounding normal tissue as possible; eight quantitative parameters were automatically generated.

Eight quantitative parameters were obtained for each RF grayscale image frame, and the parameter values of each of the three frames were averaged. Then, the average of RF quantitative parameters obtained by two sonographers was recorded as the final result and reviewed by the senior physician.

### 2.5 Statistical analysis

Statistical analyses were performed using SPSS 23.0 software (IBM SPSS INC.). Quantitative data were expressed as the mean ± standard deviation (SD), and count data were statistically described using number of cases and rate. The chi‐square test and independent samples *t*‐test were used for one‐way analysis. For indicators with *p* < 0.05 in the univariate analysis, the area under the ROC curve (AUC) was used to evaluate the diagnostic efficacy of the quantitative RF parameters and TI‐RADS. Indicators with AUC >0.50 were subjected to linear regression analysis to compare the correlation between quantitative RF parameters and TI‐RADS with CLNM using standard coefficients.

## 3 RESULTS

### 3.1 Basic information on CLNM

Of 170 patients and 182 nodes included in the study, 11 had multiple nodes (10 cases with two nodes and one case with three nodes). There were 36 men and 134 women aged 14–72 years (mean, 44.59 ± 12.56 years); tumor maximum diameter ranged from 3.5 to 63.0 mm (mean, 9.69 ± 7.50 mm). According to the number of patients, there were 68 cases (40.0%) in the positive group with CLNM and 102 cases (60.0%) in the negative group without CLNM. According to the number of nodes, there were 74 cases (40.7%) in the positive group with CLNM and 108 cases (59.3%) in the negative group without CLNM. After confirmation by preoperative puncture biopsy, 27 patients underwent lateral cervical lymph node dissection.

### 3.2 Univariate analysis of CLNM

Comparison of gender, age, and ultrasound measurements between the positive and negative groups showed significant differences in age (*t* = 2.104, *p* = 0.037), tumor maximum diameter (*t* = −4.361, *p* < 0.001), cross‐sectional aspect ratio (*t* = 2.740, *p* = 0.007), and longitudinal aspect ratio (*t* = 3.036, *p* = 0.003). There were no statistically significant differences in gender, age (≥55 years/<55 years), and tumor volume between the two groups (Table 1).

**TABLE 1:** Univariate analysis of gender, age, and ultrasound measurements with CLNM in PTC.

Variable	Positive group(*n* = 68)	Negative group (*n* = 102)	Statistics	*p*
Gender (male/female)	14/54	22/80	*x* ^2^ = 0.023	*p* = 0.878
Age	42.1 ± 13.2	46.2 ± 11.9	*t* = 2.104	*p* = 0.037
Age(≥55 years/<55 years)	12/56	26/76	*x* ^2^ = 1.446	*p* = 0.229

Variable	Positive group(*n* = 74)	Negative group(*n* = 108)	Statistics	*p*
Tumor maximum diameter (mm)	12.5 ± 9.5	7.8 ± 4.9	*t* = −4.361	*p* < 0.001
Tumor volume (ml)	2.1 ± 9.7	0.4 ± 1.7	*t* = −1.812	*p* = 0.072
Cross‐sectional aspect ratio	1.0 ± 0.2	1.1 ± 0.3	*t* = 2.740	*p* = 0.007
Longitudinal aspect ratio	0.9 ± 0.2	1.0 ± 0.3	*t* = 3.036	*p* = 0.003

**FIGURE 3:**
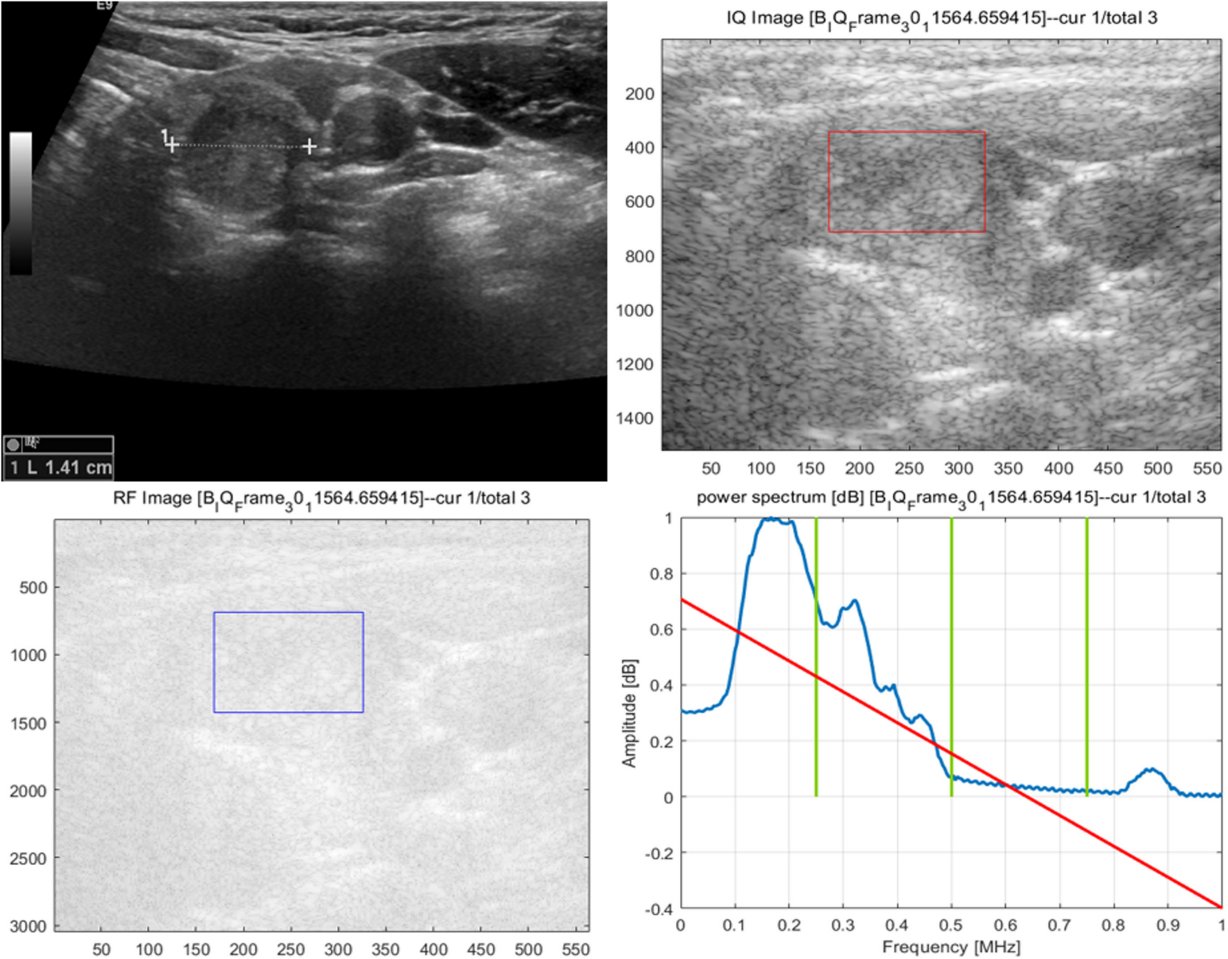
Male 50 years, left PTC with CLNM, 13.2 × 13.5 × 14.1 mm in size, solid, hypoechoic, irregular margin with punctate echogenic foci.

**FIGURE 4:**
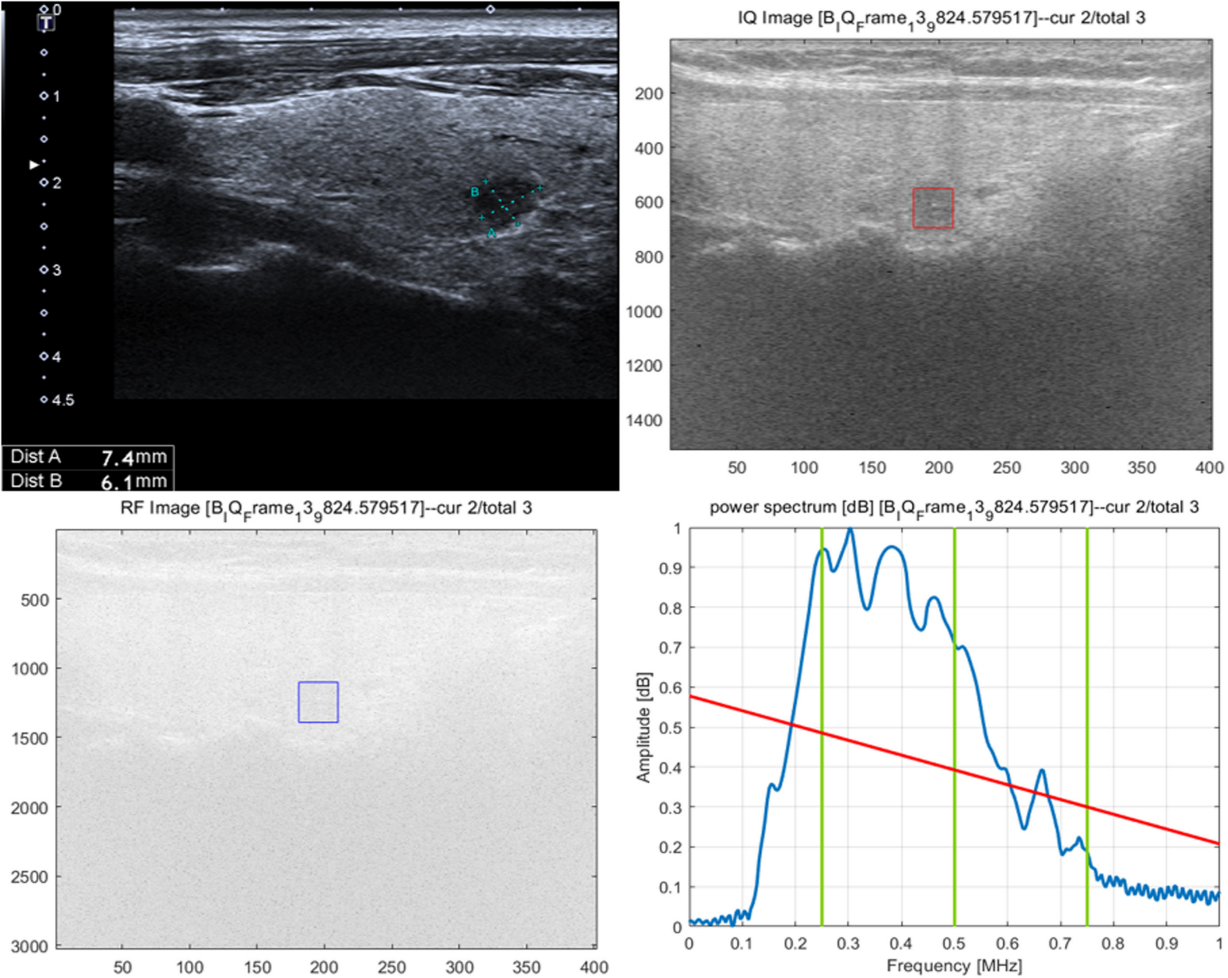
Female 45 years, right PTMC without CLNM, 7.4 × 6.1 × 6.5 mm in size, solid, hypoechoic, ill‐defined margin with punctate echogenic foci.

According to the quantitative RF signal parameters, significant differences between the two groups were observed in the transverse intercept (*t* = 2.349, *p* = 0.020), transverse mid‐band (*t* = 2.455, *p* = 0.015), transverse S1 (*t* = 2.123, *p* = 0.035), transverse S4 (*t* = 2.222, *p* = 0.028), longitudinal Higuchi (*t* = 2.969, *p* = 0.003), longitudinal slope (*t* = −2.299, *p* = 0.023), longitudinal intercept (*t* = 3.182, *p* = 0.002), longitudinal mid‐band (*t* = 2.203, *p* = 0.029), and longitudinal S1 (*t* = 2.131, *p* = 0.034). There were no statistically significant differences in transverse Higuchi, slope, S2, S3 and longitudinal S2, S3, and S4 between the two groups (Table 2, Figures 3 and 4).

**TABLE 2:** Univariate analysis of the quantitative RF signal parameters with CLNM in PTC.

Variable		Positive group(*n* = 74)	Negative group(*n* = 108)	Statistics	*p*
Transverse	Higuchi	2.61 ± 0.13	2.63 ± 0.12	*t* = 1.004	*p* = 0.317
	Slope	−0.52 ± 0.13	−0.55 ± 0.12	*t* = −1.484	*p* = 0.139
	Intercept	0.64 ± 0.04	0.65 ± 0.05	*t* = 2.349	*p* = 0.020
	Mid‐band	0.50 ± 0.05	0.52 ± 0.07	*t* = 2.455	*p* = 0.015
	S1	0.10 ± 0.04	0.12 ± 0.04	*t* = 2.123	*p* = 0.035
	S2	0.18 ± 0.05	0.17 ± 0.05	*t* = −1.434	*p* = 0.153
	S3	0.07 ± 0.03	0.07 ± 0.03	*t* = −0.834	*p* = 0.405
	S4	0.02 ± 0.01	0.03 ± 0.02	*t* = 2.222	*p* = 0.028
Longitudinal	Higuchi	2.58 ± 0.13	2.63 ± 0.11	*t* = 2.969	*p* = 0.003
	Slope	−0.50 ± 0.13	−0.55 ± 0.12	*t* = −2.299	*p* = 0.023
	Intercept	0.63 ± 0.04	0.65 ± 0.05	*t* = 3.182	*p* = 0.002
	Mid‐band	0.49 ± 0.05	0.51 ± 0.07	*t* = 2.203	*p* = 0.029
	S1	0.10 ± 0.04	0.11 ± 0.04	*t* = 2.131	*p* = 0.034
	S2	0.18 ± 0.05	0.17 ± 0.05	*t* = −1.292	*p* = 0.198
	S3	0.07 ± 0.03	0.07 ± 0.03	*t* = −1.100	*p* = 0.273
	S4	0.02 ± 0.01	0.03 ± 0.01	*t* = 1.196	*p* = 0.233

Based on ultrasonographic features, the results showed a significant difference in echogenic foci (*x* ^2^ = 11.775, *p* = 0.008), whereas location, echogenicity, aspect ratio, margin, blood flow rate, and elasticity score did not differ significantly between the two groups (Table 3).

**TABLE 3:** Univariate analysis of ultrasonographic features with CLNM in PTC.

Variable	Positive group(*n* = 74)	Negative group(*n* = 108)	Statistics	*p*
Location			*x* ^2^ = 5.575	*p* = 0.472
Left upper	6 (8.1)	6 (5.5)		
Left middle	19 (25.7)	34 (31.5)		
Left lower	8 (10.8)	10 (9.3)		
Right upper	9 (12.2)	9 (8.3)		
Right middle	16 (21.6)	35 (32.4)		
Right lower	12 (16.2)	11 (10.2)		
Isthmus	4 (5.4)	3 (2.8)		
Echogenicity			*x* ^2^ = 1.386	*p* = 0.239
Hypoechoic	74 (100.0)	106 (98.1)		
Very hypoechoic	0 (0)	2 (1.9)		
Aspect ratio			*x* ^2^ = 1.045	*p* = 0.307
<1	28 (37.8)	33 (30.6)		
≥1	46 (62.2)	75 (69.4)		
Margin			*x* ^2^ = 1.093	*p* = 0.296
Smooth or ill‐defined	43 (58.1)	71 (65.7)		
Lobulated or irregular	31 (41.9)	37 (34.3)		
Echogenic foci			*x* ^2^ = 11.775	*p* = 0.008
None or large comet‐tail artifacts	21 (28.4)	57 (52.8)		
Marco	10 (13.5)	9 (8.3)		
Peripheral	0 (0)	1 (0.9)		
Punctate echogenic foci	43 (58.1)	41 (38.0)		
Blood flow rate			*x* ^2^ = 5.147	*p* = 0.273
0	36 (48.6)	65 (60.2)		
1	22 (29.7)	32 (29.6)		
2	11 (14.9)	8 (7.4)		
3	4 (5.4)	2 (1.9)		
4	1 (1.4)	1 (0.9)		
Elasticity score			*x* ^2^ = 1.211	*p* = 0.750
2	0 (0)	1 (0.9)		
3	35 (47.3)	56 (51.8)		
4	37 (50.0)	49 (45.4)		
5	2 (2.7)	2 (1.9)		

### 3.3 ROC analysis for independent variables associated factors

For the indicators with *p* < 0.05 in the univariate analysis, ROC curves were generated for age, ultrasound measurements, quantitative RF parameters, and TI‐RADS. The AUC for tumor maximum diameter was 0.68, with specificity of 73.1% and sensitivity of 60.8%; the AUC for longitudinal slope was 0.61, with specificity of 67.6% and sensitivity of 55.4%; and the AUC for echogenic foci was 0.62, with specificity of 62.0% and sensitivity of 58.1% (Table 4).

**TABLE 4:** ROC analysis of the independent variables with CLNM in PTC.

Variable	AUC	95% CIs	*p*	Specificity	Sensitivity
Age	0.40	0.32 – 0.49	*p* = 0.033	95.1%	7.4%
Tumor maximum diameter	0.68	0.60 – 0.76	*p* < 0.001	73.1%	60.8%
Cross‐sectional aspect ratio	0.40	0.32 – 0.48	*p* = 0.021	3.7%	95.9%
Longitudinal aspect ratio	0.38	0.30 – 0.47	*p* = 0.008	0.9%	98.6%
Transverse					
Intercept	0.42	0.34 – 0.50	*p* = 0.068	21.3%	83.8%
Mid‐band	0.41	0.33 – 0.50	*p* = 0.049	2.8%	97.3%
S1	0.41	0.33 – 0.49	*p* = 0.043	7.4%	93.2%
S4	0.45	0.36 – 0.53	*p* = 0.230	29.6%	73.0%
Longitudinal					
Higuchi	0.39	0.31 – 0.47	*p* = 0.012	1.9%	95.9%
Slope	0.61	0.52 – 0.69	*p* = 0.016	67.6%	55.4%
Intercept	0.39	0.30 – 0.47	*p* = 0.008	0.9%	98.6%
Mid‐band	0.42	0.33 – 0.50	*p* = 0.057	4.6%	93.2%
S1	0.39	0.30 – 0.47	*p* = 0.009	0.9%	95.9%
Echogenic foci	0.62	0.54 – 0.71	*p* = 0.005	62.0%	58.1%

### 3.4 Linear regression analysis of independent variables

Linear regression analysis was performed for factors with AUC >0.50 to compare the correlation between ultrasound measurements, quantitative RF parameters, and TI‐RADS with CLNM by standard coefficients. Tumor maximum diameter had the greatest correlation with CLNM (*ß* = 0.285), and longitudinal slope had a greater correlation with CLNM than echogenic foci (*ß* = 0.203 vs. *ß* = 0.154) (Table 5).

**TABLE 5:** Linear regression analysis of the independent variables with CLNM in PTC.

Model	Denormalization coefficient		Standardization coefficient	Statistics	*p*
	*B*	Standard error	*ß*		
(Constant)	1.571	0.152		*t* = 10.304	*p* < 0.001
Tumor maximum diameter	0.019	0.005	0.285	*t* = 3.973	*p* < 0.001
Longitudinal slope	0.803	0.273	0.203	*t* = 2.939	*p* = 0.004
Echogenic foci	0.053	0.025	0.154	*t* = 2.148	*p* = 0.033

## 4 DISCUSSION

Thyroid cancer is a common endocrine malignancy, and PTC is the most common pathological type; it is characterized by low‐grade malignant growth and low rates of distant metastasis and mortality; however, it is prone to CLNM.^21^ Although ultrasonography is the primary screening method for the thyroid gland, grayscale images of benign and malignant thyroid nodules overlap, and the correlation with CLNM is not conclusive. Therefore, identifying more objective and standardized covariates is necessary to expand the clinical applications of ultrasonography.

PTC can occur at all ages, although tumorigenesis and the presence of suspicious lymph nodes are more common at age < 40 years than at age ≥ 40 years (60.6% vs. 37.7%).^24^ The mean age of the positive group in this study was significantly lower than that of the negative group (*p* < 0.05). The risk of PTC with CLNM has a positive linear relationship with the size of the primary focus.^25^ The results of this study showed that the tumor maximum diameter of the positive group was significantly larger than that of the negative group (*p* < 0.001). The AUC of tumor maximum diameter was 0.68, with specificity and sensitivity of 73.1% and 60.8%, respectively. Linear regression analysis showed the greatest correlation between tumor maximum diameter and CLNM, which was consistent with the results of previous studies.^26^, ^27^

Aspect ratio ≥1 was a highly specific indicator for the diagnosis of malignant thyroid nodules.^28^, ^29^ Studies^26^, ^30^ show that aspect ratio >1 is associated with a higher likelihood of CLNM. In this study, cross‐sectional and longitudinal aspect ratio according to tumor morphology were statistically significant in the univariate analysis (*p* < 0.05); however, the AUC values were <0.50 for both, which may be related to the low number of cases enrolled. A previous study by our group^10^ showed that for smaller PTC lesions, the cross‐sectional aspect ratio has a better predictive value for CLNM. Echogenic foci reflect gravel bodies in the pathology and are significantly associated with CLNM.^31^ In this study, the AUC of echogenic foci was 0.62, supporting that it could be an important risk factor for predicting the risk of CLNM in PTC.

Compared with post‐processed gray‐scale images, the RF signal retains complete echo information and could be used to extract and analyze the microstructural features of scattering within the tissue.^32^ The common parameters reported in the literature^33^ are entropy, weighted entropy, Nakagami‐m, and kurtosis. This study used different RF parameters with the objective of introducing new quantitative parameters to obtain a more stable and reliable basis for evaluation.

Slope corresponds to the characteristics of scatter size, and it is an important indicator of tissue microstructure. In this study, longitudinal slope differed significantly between the positive and negative groups (*p* < 0.05), and the AUC was 0.61, which was comparable to the AUC of 0.62 for echogenic foci. Linear regression analysis showed that the correlation between the longitudinal slope and CLNM was greater than that of echogenic foci. Further studies with a greater number of cases are needed to determine whether longitudinal slope can assume an important role in the interpretation of RF signals.

Higuchi describes textural characteristics such as complexity, roughness, and irregularity in the ROI region, whereas intercept and mid‐band correspond to the tissue characteristics such as density and acoustic impedance; S1, S2, S3, and S4 represent the low, medium‐low, medium‐high, and high frequency components of the RF signal, respectively. In this study, significant differences were observed in transverse intercept, mid‐band, S1, and S4 and longitudinal Higuchi, intercept, mid‐band, and S1 (*p* < 0.05) in the univariate analysis; however, the AUC values were all <0.50, and the diagnostic efficacy for predicting CLNM was low.

Ultrasonic RF signals are raw data that may correspond to thousands of parameters, and studies involving a greater number of cases and research centers are necessary to extract meaningful indicators for clinical diagnosis from the complex data. Expert consensus regarding the RF signals is necessary for the application of RF signal parameters, and it can effectively promote the application of RF signal parameters. In this study, we did not apply AI to deep learning because of the small number of cases, and the study was a preliminary exploration of quantitative RF signal parameters. RF signals combined with artificial neural networks may achieve 93.2% sensitivity, 94.0% specificity, and 93.5% accuracy in the classification of benign and malignant thyroid nodules.^33^ RF signals can be used to establish a standardized image database, and the combination with AI should be the future of imaging technology. RF quantitative parameters can play an important role in ultrasound imaging omics and ultrasound AI, and it can be combined with ultrasound equipment in the future to optimize screening for high‐risk individuals with PTC.

The present study had several limitations as follows: (1) this was a single‐center retrospective study with a small number of cases, and the enrolled cases were all confirmed PTC, which may cause selective bias; (2) 93.5% of the included subjects had unifocal disease, and the association of multifocal disease, different regions of the neck, symptoms, thyroid biochemical indicators and pathological subtypes with lymph node metastasis could not be assessed; (3) the combination of quantitative RF parameters, RF signals, and AI needs to be validated by prospective studies including large amounts of data and multicenter follow‐up research.

In conclusion, quantitative RF parameters have good reference value for the preoperative assessment of CLNM. Longitudinal slope and echogenic foci showed similar diagnostic efficacy for predicting CLNM in PTC, although the correlation between longitudinal slope and CLNM was greater. Further research on quantitative RF parameters is necessary. RF signals could be an effective assessment indicator that can be widely used in the field of thyroid nodule diagnosis and treatment.

## AUTHOR CONTRIBUTIONS

**Liuhua Zhou:** Data curation (equal); software (equal); writing – original draft (lead). **Yi Zheng:** Data curation (equal); software (equal); writing – original draft (supporting). **Jincao Yao:** Data curation (supporting); software (supporting). **Liyu Chen:** Data curation (equal); methodology (supporting); supervision (supporting). **Dong Xu:** Conceptualization (lead); funding acquisition (lead); project administration (lead); resources (lead); writing – review and editing (lead).

## FUNDING INFORMATION

This study was supported by National Natural Science Foundation of China (No. 82071946) and Pioneer and Leading Goose R&D Program of Zhejiang (No. 2023C04039). DX is responsible for these two funds.

## CONFLICT OF INTEREST STATEMENT

We declare that no authors are involved in conditions that may influence our professional judgment concerning the validity of research, and we were not influenced by financial gain. Data and material are available and reliable in this article.

## ETHICS STATEMENT

This study was approved by the Ethics Committee of the Cancer Hospital of the University of Chinese Academy of Sciences (Zhejiang Cancer Hospital). All methods were carried out in accordance with relevant guidelines and regulations. Written informed consent was obtained from all patients, or from a parent or guardian for participants under 16 years of age.

## CONSENT FOR PUBLICATION

The scientific guarantor of this publication is Professor Dong Xu. Permission has been received that any material in the article can be used, and consent for publication has been acquired from all authors.

## Data Availability

The datasets used and analyzed during the study are available from the corresponding author upon reasonable request.
